# Observation of the geometric phase effect in the H+HD→H_2_+D reaction below the conical intersection

**DOI:** 10.1038/s41467-020-17381-4

**Published:** 2020-07-20

**Authors:** Daofu Yuan, Yin Huang, Wentao Chen, Hailin Zhao, Shengrui Yu, Chang Luo, Yuxin Tan, Siwen Wang, Xingan Wang, Zhigang Sun, Xueming Yang

**Affiliations:** 1 Hefei National Laboratory for Physical Sciences at the Microscale and Department of Chemical Physics, University of Science and Technology of China, Hefei, 230026 China; 2 State Key Laboratory of Molecular Reaction Dynamics, Dalian Institute of Chemical Physics, Chinese Academy of Sciences, Dalian, 116023 China; 3 Hangzhou Institute of Advanced Studies, Zhejiang Normal University, Hangzhou, 311231 China; 4 Department of Chemistry, College of Science, Southern University of Science and Technology, Shenzhen, 518055 China

**Keywords:** Reaction kinetics and dynamics, Reaction mechanisms

## Abstract

It has long been known that there is a conical intersection (CI) between the ground and first excited electronic state in the H_3_ system. Its associated geometric phase (GP) effect has been theoretically predicted to exist below the CI since a long time. However, the experimental evidence has not been established yet and its dynamical origin is waiting to be elucidated. Here we report a combined crossed molecular beam and quantum reactive scattering dynamics study of the H+HD → H_2_+D reaction at 2.28 eV, which is well below the CI. The GP effect is clearly identified by the observation of distinct oscillations in the differential cross section around the forward direction. Quantum dynamics theory reveals that the GP effect arises from the phase alteration of a small part of the wave function, which corresponds to an unusual roaming-like abstraction pathway, as revealed by quasi-classical trajectory calculations.

## Abstract (web summary)

The geometric phase effect associated with a conical intersection between the ground and first excited electronic state has been predicted in the H_3_ system below the conical intersection energy. The authors, by a crossed molecular beam technique and quantum dynamic calculations, provide experimental evidence and insight into its origin.

## Introduction

The geometric phase (GP) is an important concept in adiabatic theory. It represents the phase acquired when a quantum state is adiabatically transported around a closed circuit in the parameter space, while remaining in the same Hilbert space. The concept of the GP has given rise to various observable effects, and has now become a central topic across various research fields ranging from chemistry to condensed matter physics^1,2^.

A classical example of GP in condensed phase physics is the anomalous Hall effect^3^. In a molecule, the presence of a conical intersection (CI) leads to the appearance of the GP and enables rapid conversion of the excess electronic energy into nuclear motion. The GP induces a sign change of the adiabatic electronic wave functions along a closed path of the nuclear configuration space encircling the CI^4–7^. For a molecular system with a CI, the theoretical treatment in the adiabatic picture requires the inclusion of the GP to make sure that at each nuclear geometry, the total wave function can be single-valued, although the nuclear wave function is localized away from the region of the CI. The GP effect in the spectra of molecular vibrational bound states has been well studied with Jahn–Teller molecules^8–10^. Recently, the significant role of the GP in photodissociation process of phenol has been reported^7,11,12^. In larger molecules, the increased number of degrees of freedom and electronic states leads to the general presence of CIs in various molecular photoinduced chemical dynamics processes involving electronically excited states, where CIs play a very important role^13^.

H+H_2_ is one of the most important chemical reactions since it can be accurately described with current quantum theoretical methods. It exhibits a typical *D*_3h_ CI between the ground and first excited electronic states at equilateral triangle geometries, corresponding to the total energy of about 2.75 eV. Using the ground adiabatic potential energy surface (PES), product state-resolved integral and differential cross sections (DCS) for the H+H_2_ reaction and its isotopologues without consideration of the GP were first reported in 1988^14,15^. Later, it was found that the adiabatic quantum dynamics predictions without the GP (i.e. NGP) agree with experiments at some collision energies quite well, suggesting that the GP may not be important in this reaction and its isotopologues^16–21^. Mead and Truhlar found that the GP would only influence observables when the nuclear wavefunction encircled the CI, and the GP effect could be involved by the introduction of a vector potential^22^.

In the last decades, a series of quantum mechanical theoretical studies on the H+H_2_ reactive scattering were carried out by Kendrick and by Althorpe and coworkers. Their results demonstrated that the GP effect should be negligible at total energies below 1.6 eV^23–26^, and only becomes significant when the total energy is above 3.5 eV. Quantum wave-packet results by Althorpe and coworkers also suggested that a shift of the fast oscillatory angular distributions of the sideways scattered products could serve as a clear signature to reveal the effect of GP^19,27,28^. Recently, Kendrick and his coworkers found that the GP effectively controls the reactivity of the H+H_2_ (*v* = 4, *j* = 0) and OH+O → O_2_+H reactions in ultracold conditions^29,30^.

In the past several decades, high-resolution crossed molecular beam (CMB) studies have been carried out on many important elementary reactions using the Rydberg state H-atom tagging method^31,32^, including the H+HD and H+D_2_ reactions at a set of different collision energies^16,18,20,33^. No fast angular oscillations in DCS were observed, and this is most probably due to the limited experimental angular resolution. More recently, the PHOTOLOC technique has been employed in the study of the GP effect in the H+H_2_ reaction and its related isotopologues, but no clear evidence of the GP was found^34,35^.

Very recently, with a high-resolution time-sliced velocity map imaging (VMI) – CMB apparatus, we detected D products of the H+HD reactions by using the near threshold ionization scheme^21,36^. The product ro-vibrational state-resolved DCS with fast oscillatory structures in the forward angular distributions have been observed at the collision energies of 1.35 and 2.77 eV^21,36^. At 1.35 eV, which is far below the CI, the reaction appears to take place via a simple direct abstraction mechanism, where the GP plays a negligible role. At 2.77 eV (about 2.99 eV in total energy relative to the equilibrium energy of the H_2_ molecule), which is 0.24 eV above the CI, the measured fast angular oscillations of the forward scattered products in the product ro-vibrational state-resolved DCS could only be reproduced by quantum mechanical calculations on the lowest adiabatic PES with GP included, clearly demonstrating the existence of the GP effect in the reaction.

In previous work^36^, quantum dynamics calculations using accurate diabatic coupled PESs had to be carried out to exclude the role of the adiabatic electronically excited state since the studied energy was above the CI. This follows an intriguing question: at an energy well below the CI, where the role of the adiabatic electronically excited state could be simply excluded, will there be observable effects due to the GP?

Here we report a high-resolution CMB study on the H+HD → H_2_+D reaction at the collision energy of 2.28 eV, corresponding to 2.50 eV in total energy, or 0.25 eV below the CI. In addition, we carry out accurate adiabatic quantum mechanical time-dependent wave packet calculations with and without considering the GP effect in hyperspherical coordinates to investigate the role of GP on this reaction^37–39^. The GP effect is analyzed by calculating the time-independent scattering wave functions at 2.28 eV in hyperspherical coordinates. At the same time, the dynamical mechanism determining the GP effect is revealed using quasi-classical trajectory (QCT) calculations.

## Results

### High-resolution experimental image

**Fig. 1:**
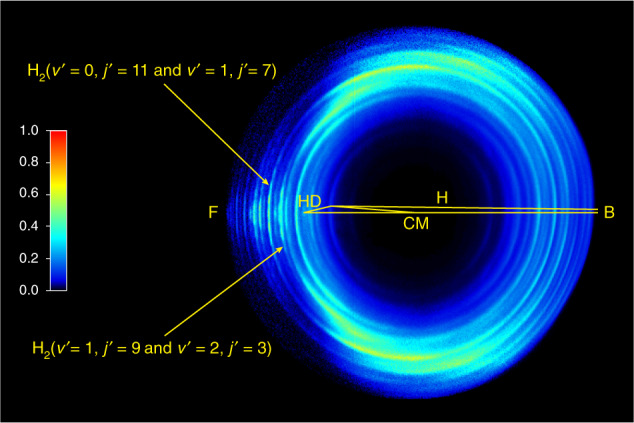
Experimental image. The experimental image of the D product from the H+HD → H_2_+D reaction at 2.28 eV. “H”, “HD” and “CM” denote the velocity vectors of H beam, HD molecular beam and the origin of center of mass frame, respectively, relative to the origin of the laboratory coordinate system. The crossing angle of the H and HD beams is set to 160°. The forward (0°) and the backward (180°) scattering directions for the H_2_ coproduct are defined in the center-of-mass frame relative to the H-atom beam direction, and are denoted as “F” and “B” in the image, respectively. The ro-vibrational states of H_2_ products are pointed out by arrows and labeled as H_2_ (*v*′ = 0, *j*′ = 11 & *v*′ = 1, *j*′ = 7) and H_2_ (*v*′ = 1, *j*′ = 9 & *v'* = 2, *j*′ = 3). The normalized intensity of the image is defined by the color scale on the left of the image.

**Fig. 2:**
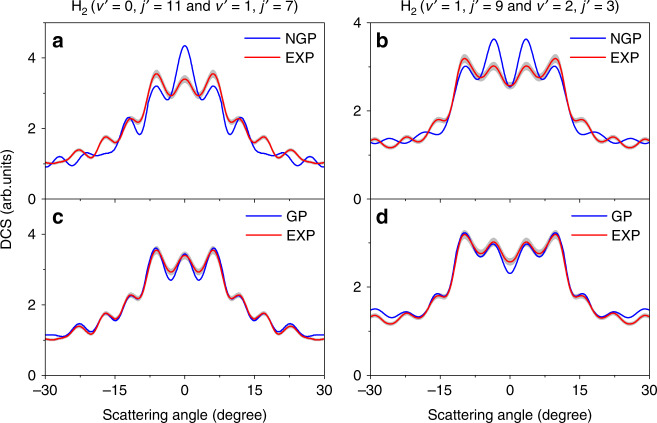
Product angular distributions. The experimental (EXP) and theoretical (NGP and GP) DCSs in the forward scattering direction for the H_2_ products in the H+HD (*v* = 0, *j* = 0) → H_2_+D reaction at 2.28 eV. **a** and **c** Product ro-vibrational states are (*v*′, *j*′) = (0, 11) & (1, 7); **b** and **d** Product ro-vibrational states are (*v*′, *j*′) = (1, 9) & (2, 3). “&” connects two ro-vibrational states with very small energy differences, that correspond to the rings labeled by yellow arrows in the image (see Fig. 1 and Supplementary Fig. 3). The experimental errors standard deviation (SD) are displayed in gray. The theoretical results (blue lines) in panels **a** and **b** do not include the geometric phase (NGP), while the theoretical results (blue lines) in panels **c** and **d** do include it (GP).

The H+HD→H_2_+D reactive scattering experiment is carried out using a CMB apparatus with a VMI detector at the collision energy of 2.28 eV. A more detailed experimental procedure is presented in the Supplementary Note 1. The experimental image of the D product (Fig. 1) exhibits a series of well-resolved rings coming from different ro-vibrational states of the H_2_ product, which are clearly assignable. Fine oscillatory features in the forward angular distributions are successfully captured, similar to those observed at 1.35 eV and 2.77 eV^21,36^. There are two very pronounced rings labeled by yellow arrows in Fig. 1. Each of them corresponds to two ro-vibrational states with very small energy differences. The experimental angular distributions in the forward scattering direction are presented in Fig. 2 for the H_2_ product at the (*v*′ = 0, *j*′ = 11 & *v*′ = 1, *j*′ = 7) and (*v*′ = 1, *j*′ = 9 & *v*′ = 2, *j*′ = 3) levels. Here “&” is used to connect the two ro-vibrational states with very small energy differences. For the corresponding rings, the angular distributions are derived by extracting the signals at various scattering angles (refer to Supplementary Note 2).

### GP identification via quantum dynamics calculations

In order to understand the reaction dynamics of the H+H_2_ reaction and its isotopologues, recently we have developed a state-to-state time-dependent quantum reactive scattering theory in hyperspherical coordinates with and without the inclusion of the GP as a vector potential, similar to that in the Jacobi coordinates^25,36^. In hyperspherical coordinates, it is straightforward to express the vector potential for the CI of *D*_3h_, which is the function of only the two hyperangles *θ* and *χ*, thus it is convenient to analyze the GP effect in the reaction with wave functions in hyperspherical coordinates.

To justify whether there is a detectable GP effect at this energy for the H+HD reaction, the adiabatic quantum dynamics calculations are first carried out on the accurate adiabatic BKMP2 PES without including the GP effect (NGP) (Fig. 2a, b). The DCS from the NGP calculations clearly deviated from the experimental results. Especially for the plot in Fig. 2b, the slight oscillations in the 10°–30° angular range of the experimental angular distribution for the H_2_ (*v*′ = 1, *j*′ = 9 & *v*′ = 2, *j*′ = 3) product states are nearly completely out-of-phase with the theoretical NGP DCS, with the peaks in the calculated DCS located at the valley positions of the experimental one.

Time-dependent adiabatic quantum dynamics calculations are then carried out for the H+HD → H_2_+D reaction on the BKMP2 PES. A vector potential is introduced to include the effect of GP. The calculated DCS with the GP included are shown in Fig. 2c, d. The theoretical GP DCS is in excellent agreement with the experimental results, with the theoretical angular oscillations in the DCS right in phase (and amplitude) with the experimental results, while the NGP results show clear differences with the experimental one. This suggests that, in the adiabatic picture, the GP effect can be unambiguously observed for the benchmark reaction at an energy well below the CI.

## Discussion

At the current relative low collision energy, it is impressive that the GP effect on the H+HD reaction can be identified so clearly around the forward direction in the DCS. The GP only bring in a phase change to the wave functions. The topological argument^25^ therefore infers that the reaction dynamics will not be influenced by the GP if the reaction only take place via a single pathway. As a result, a second reaction pathway, besides the normal abstraction one, is expected for the H+HD → H_2_+D reaction at 2.28 eV, similar to that for the H+H_2_ reaction. The GP can then alter the interference pattern between these two pathways thus leading to variations in the pattern of the DCS of the products.

In order to investigate the mechanisms accounting for the GP effect in the H+HD → H_2_+D reaction, we have calculated the time-independent scattering wave function as

1 $${\mathrm{\Psi }}\left( {\rho ,\theta ,\chi ;E_0} \right) = \int_{ - \infty }^{ + \infty } {{\mathrm{\Psi }}\left( {\rho ,\theta ,\chi ;t} \right)e^{i(E_c + E_0)t}dt},$$

with *E*_c_ = 2.28 eV and *E*_0_ as the zero point energy. At *ρ* ~ 2.5 bohr, where the CI has the lowest energy, the GP is expected to play the most significant role. Thus, a closer examination of the wave function at *ρ* ~ 2.5 bohr would be helpful for understanding the mechanisms.

**Fig. 3:**
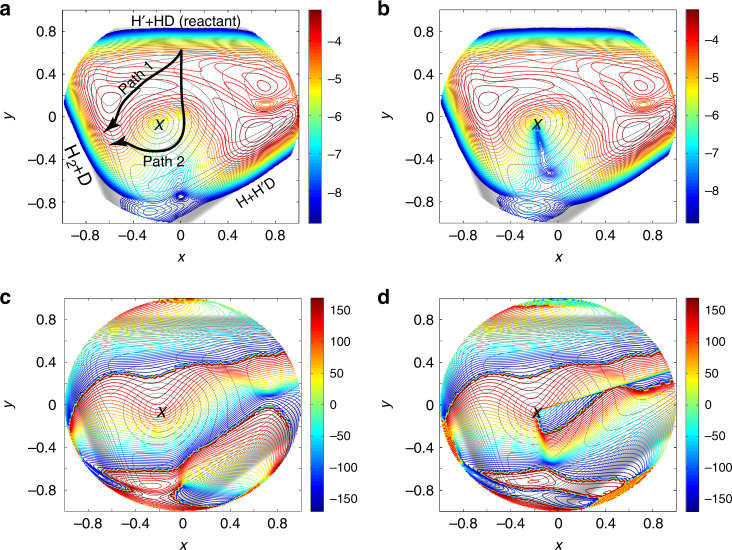
Wavefunction analysis of the GP effect. **a** The scattering wave function|***ψ***(*ρ, θ, χ; E*_c_)|^2^ in logarithmic scale (base 10) of the NGP calculations in the H + HD → H_2_ + D reaction at 2.28 eV of adiabatic ground state at hyperradius *ρ* ≈ 2.5 bohr, where *x* = sin(*θ*)cos(2*χ*) and *y* = sin(*θ*)sin(2*χ*), along with the adiabatic ground potential energy surface. **b** Similar to that in Panel (**a**) but for the wave function of the GP calculation. The color scales on the right denote the intensities of the wave function (in logarithmic scale) in (**a**) and (**b**); **c** The phase of the wave function in (**a**) in degrees; **d** The phase of wave function in (**b**) in degrees. The color scales on the right denote the phase values in (**c**) and (**d**). “X” in the wave function patterns denotes the position of the conical intersection. “Path 1” and “Path 2” represent the reaction paths which experience one and two transition states, respectively.

The wave functions at *ρ* ~ 2.5 bohr as a function of *x* = sin(*θ*)cos(2*χ*) and *y* = sin(*θ*)sin(2*χ*) of the NGP and GP calculation are presented in Fig. 3a, b in logarithmic scale (base 10). It is not surprising that the majority part of these two wave functions, which are away from and above the CI in the figure, are almost identical. These regions correspond to the normal abstraction process from the reactant H´ + HD to products H_2_ + D and H´D + H, which experiences only one transition state. We name it as Path 1 (as shown in Fig. 3a).

However, in the region just below the CI, there is a significant difference between these two wave functions, although the amplitudes of the wave functions are quite small. In that region, the GP wave function acquires a node structure that does not appear in the NGP wave function. This part of the wave function should correspond to the reaction path which experiences two transition states^23^. We name it as Path 2 (as shown in Fig. 3a).

The phases of the wave functions are further calculated and presented in Fig. 3c, d for the NGP and GP calculations, respectively. The line, which starts from the CI and extends up to the right, results from the boundary condition of double values for the nuclear wave function introduced by the vector potential. The phases of the NGP and GP wave functions in most regions are very similar. However, in the area to the right of the node of the wave function below the CI and below the double values boundary condition line, which is mainly located in the (x > 0, y < 0) quadrant in Fig. 3c, d, the phase of the GP wave function differs by about 180° from that of the NGP wave function. This is the physical origin of the difference between the GP and NGP DCS. This additional node in the GP wavefunction in the chemical reaction is very similar to the GP wavefunction in a photodissociation process^7,12^.

Althorpe and coworkers also extracted the contributions of the two classes of reaction pathways with an approach based on topological arguments^25–27^. In their studies, the nuclear wave functions from Path 1 and Path 2 can be derived by *ψ*_1_ = $(\psi _{NGP} + \psi _{GP})/\sqrt 2$ and *ψ*_2_ =$(\psi _{NGP} - \psi _{GP})/\sqrt 2$, respectively. Subscripts “NGP” and “GP” are used to denote the wave functions without and with the introduction of the GP effect (similarly hereinafter), respectively. The scattering amplitudes from these two pathways can be expressed as,

$$f_1({\uptheta}) = [f_{{\mathrm{NGP}}}({\uptheta}) + f_{{\mathrm{GP}}}({\uptheta})]/\sqrt 2 \, \, {\mathrm{and}}\, \, f_2({\uptheta}) = [f_{{\mathrm{NGP}}}({\uptheta}) - f_{{\mathrm{GP}}}({\uptheta})]/\sqrt 2 ,$$

respectively. The DCSs for the individual paths are given by the square moduli of *f*_1_(θ) and *f*_2_(θ), |*f*_1_(θ)|^2^ and |*f*_2_(θ)|^2^. The total DCS for the reaction can be described as,

2 $${\upsigma}({\uptheta}) = \left| {f_1({\uptheta}) + f_2({\uptheta})} \right|^2 = \left| {f_1({\uptheta})} \right|^2 \, + \, \left| {f_2({\uptheta})} \right|^2 + f_1^ \ast ({\uptheta})f_2({\uptheta}) + f_1({\uptheta})f_2^ \ast ({\uptheta}),$$

whereas the interference between Path 1 and Path 2 originates from the last two crossing terms. Since the GP alters the relative phases between the two pathways, a difference in the DCS appears. With this approach, we calculate the total integral cross section (ICS) for the reaction via Path 1 and Path 2 at 2.28 eV. The reaction occurring through Path 2 amounts to only about 0.35% of the total product, which is much smaller than that at 2.77 eV (~2.3% at 2.77 eV)^36^. Thus the GP effect in the DCS at 2.28 eV is weaker and much more difficult to observe experimentally. With increasing collision energies, a large portion of the wave function encircles the CI in the clockwise rotation direction from the reactant H + HD channel to the product H_2_ + D channel, thus, the DCS of the reaction manifests a stronger GP effect.

**Fig. 4:**
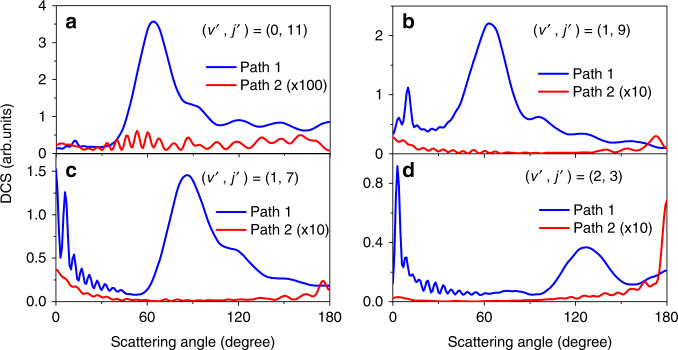
Relative differential cross sections from Path 1 and Path 2. **a** for product H_2_ (*v*′ = 0, *j*′ = 11), **b** (*v*′ = 1, *j*′ = 9), **c** (*v*′ = 1, *j*′ = 7), and **d** (*v*′ = 2, *j*′ = 3). “Path 1” and “Path 2” represent the reaction paths showed in Fig. 3a, which experience one and two transition states, respectively.

The DCS for H_2_ (*v*′ *=* 0, *j*′ = 11), (*v*′ *=* 1, *j*′ = 7), (*v*′ *=* 1, *j*′ = 9), and (*v*′ *=* 2, *j*′ = 3) from Path 1 and Path 2 at 2.28 eV are presented in Fig. 4. The results show that there are very different angular distributions for the two reaction paths. Path 1 leads to an angular distribution with the products predominantly sideways scattered compared to the relatively small forward direction, whereas Path 2 for product H_2_ (*v*′ *=* 0, *j*′ = 11) leads to oscillatory scattering evenly in the whole scattering angular range, for products H_2_ (*v*′ *=* 1, *j*′ = 7 & 9) to significant both forward and backward scattering, and for product H_2_ (*v*′ *=* 2, *j*′ = 3) to dominant backward scattering. The DCS from Path 2 is much smaller, especially for the product H_2_ (*v*′ *=* 0, *j*′ = 11), less than 1% of that from Path 1. Consequently, the oscillations around the forward scattering direction observed in Fig. 2 are weaker than those observed for product H_2_ (*v*′ *=* 0, *j*′ = 7) at the collision energy of 2.77 eV, where the DCS from the two paths are of comparable amplitude.

To shed light on the reaction mechanism about Path 2, QCT calculations are carried out using the adiabatic BKMP2 PES. As we know, the QCT theory has long been used and is able to provide an intuitive picture of the reaction mechanism^40^, although it will break down owing to the existence of quantum effects. Similar to previous work^28^, we found that the QCT theory is valid for calculating the product ro-vibrational state-resolved ICSs for both Path 1 and Path 2. This observation confirms that the QCT calculation should be credible for investigating the mechanism of Path 2 in the reaction.

**Fig. 5:**
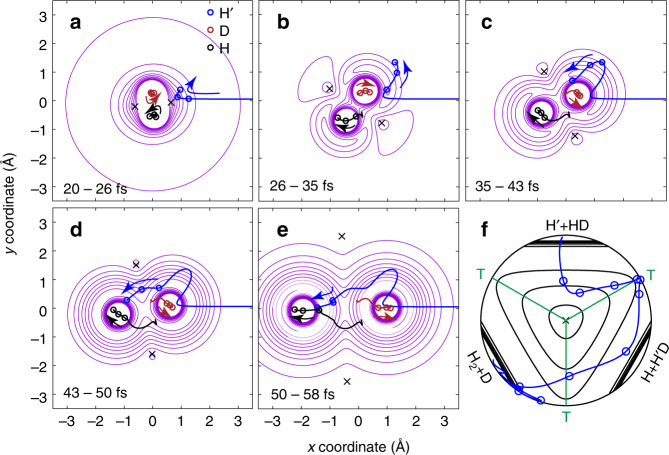
Representative trajectories. The representative trajectories for the forward scattering of the H + HD → H_2_ (*v*′ = 2, *j*′ = 3) + D reaction are presented. The incoming H atom is labeled as H′ in the figure to distinguish from the H atom in the reactant HD molecule. **a**–**e** The roaming-like abstraction reaction (Path 2) trajectory moving with time in Cartesian coordinates with forward scattering (*θ* = 5°). The blue, brown, and black curves represent the trajectories of incoming H atom, the D and H atom of the reactant HD molecule, respectively, and the blue, brown, and black arrows draw the motion direction of the corresponding atoms. The positions of the H′, D, and H atoms are plotted as blue, brown, and black circles on the contour, respectively, at a series of frames with fixed time intervals located at the center of the mass of the reactant HD molecule. The contour of the potential energy surface is at instant time 23.0, 30.5, 39.0, 46.5, and 54.0 fs for frames **a**–**e**, respectively. Crosses (×) in dark gray color denotes the location of the conical intersection. **f** The trajectory (blue line) in **a**–**e** in hyperspherical coordinates, which experiences two transition states. The dark gray cross indicates the system’s conical intersection, “T” in green represent the three transition states. and the corresponding green lines separate three different atom-diatom channels.

Similar to the previous findings with the H + H_2_ reaction^23^, the QCT results suggest that the majority of the products were generated through Path 1. However, the reaction mechanism for Path 2 is distinct from that in the H + H_2_ reaction, as the snapshots of representative trajectories for product H_2_ (*v*′ *=* 2, *j*′ = 3) shown in the movie sequence of Fig. 5a–e illustrations. In frame 20–26 fs, the hydrogen H approaches the HD molecule and enters the CI slope region^41^. Then the HD bond stretches and rotates about its center of mass with the D end toward the incoming H atom, as shown in frame 26–35 fs. At the same time, the incoming H atom is slowed by the linear well, generated by the stretch of the HD molecule^41^. In frame 35–43 fs, the incoming H atom turns around and passes by the D atom of the HD molecule. In the later frame 43–50 fs, the incoming H atom migrates toward the H atom of the HD molecule, whose bond is much stretched. Finally, as shown in frame 50–58 fs, the incoming H atom impacts at the H atom of the HD molecule to form product molecule as the HD bond breaks. The H_2_ and D products are formed, with product H_2_ scattered into the forward direction. The movie for this representative trajectory of Path 2 is provided as Supplementary Movie 1 for a better understanding. The trajectory is also plotted in hyperspherical coordinates in panel f, where it is seen clearly that the reaction by Path 2 experiences two transition states, as that in the H + H_2_ reaction^23^. One may compare the trajectory in panel f with the corresponding part of the wave function in Fig. 3. It is noted that for the product H_2_ in other ro-vibrational states, the trajectories are very similar.

This representative trajectory shares some features of the roaming phenomenon in a photodissociation process^42^: The incoming H atom first migrates around the D atom of HD for some time, but finally impacts the H atom to form the final H_2_ molecule product. Whereas there is only one simple barrier and no any effective potential well in the PES of the reaction, the migration process is transient and lasts for only about 40 fs. Therefore, we name this mechanism for Path 2 as “roaming-like” abstraction. This roaming-like reaction accounts for nearly 100% of the trajectories of Path 2, and some of them are more like insertion reaction and lead to backward scattering, in contrast to the direct insertion mechanism previously found to account for more than 90% of Path 2 in the H + H_2_ reaction.

In summary, this work clearly confirms that the GP effect does exist in the quantum dynamics of chemical reactions at energy well below the CI. The dynamical reason for the existence of the GP effect is arising from the roaming-like abstraction reaction pathway, in addition to the usual direct abstraction reaction pathway, and this roaming-like reaction accounts for nearly 100% of the trajectories of Path 2. In order to accurately describe the reaction involving CI in the adiabatic picture, one has to introduce the vector potential or similar theoretical methods to account for the GP, although the energy is well below the CI. The high-resolution VMI technique is powerful enough for observing the angular oscillations in the product state-resolved DCS, and this opens a door for studying chemical reactions within quantum mechanical principles.

## Methods

### Experimental setup

The CMBs apparatus is equipped with three differentially pumped vacuum chambers: a fixed and a rotatable source chamber, together with a scattering chamber. A high-vacuum condition (10^−7^ ~ 10^−8^ torr with two molecular beams on) can be achieved in the detection area. In this H + HD→H_2_ + D reaction, the H atom beam is produced by dissociating the supersonically expanded HI molecules with a 213 nm laser light. The supersonic HD molecular beam is generated by a liquid nitrogen cooled pulsed valve (Even-Lavie valve). In the HD beam, about 97% of the HD molecules are populated in the (*v* = 0, *j* = 0) level. During the scattering experiment, the pulsed H atom and HD molecule beams collide at a crossing angle of 160°. The D atom products are detected by a two-color (1+1′) (vacuum ultraviolet + ultraviolet) threshold ionization method. The ionized D atom fragments are guided and accelerated by the ion optics^43^ and then detected by a velocity map ion imaging device. For more experimental details, please refer to the Supplementary Note 1. For the experimental statistical error analysis, please refer to Supplementary Note 2.

### Theories

In the quantum dynamics calculations of the H + HD (*v* = 0, *j* = 0) reaction, the product quantum state-resolved *S* matrices are calculated by time-dependent wave packet method on the BKMP2 PES. The wave packet propagation is carried out in hyperspherical coordinates with the second-order split operator^37^. When the GP effects are included, a vector potential can be conveniently added to the Hamiltonian operator, in a way similar to our previous work using the reactant Jacobi coordinate^36^. In the calculations, numerical parameters (listed in Supplementary Note 3) can give converged DCS with collision energies up to 4.0 eV.

The QCT calculations for the this reaction are implemented on the BKMP2 PES^44–47^. In the calculations, a total of 23.5 million trajectories are propagated at 2.28 eV translational energy giving an overall statistical error of 0.07% in the total reactive cross section. All reactive trajectories can be classified into “Path 1” and “Path 2” paths by accounting how many times each trajectory crossed the transition states, and it is conveniently executed in the hyperspherical coordinate system. In total, 1.892 million trajectories are found to be reactive giving H_2_ product. Of these, ~99.65% reacted through one transition state (the usual direct abstraction mechanism) and only ~0.35% (6661 trajectories) through two transition states.

## Supplementary information

Supplementary Information

Description of Additional Supplementary Files

Supplementary Movie 1

## Data Availability

Data supporting the findings of this study are available from the corresponding authors upon request.

## Code availability

The code that supports the theoretical calculations in this paper is available from the corresponding authors upon request.
