# Supplementary material for: Observation of the geometric phase effect in the H+HD→H2+D reaction below the conical intersection
Source: Nat Commun. 2020 Jul 20;11:3640. doi: 10.1038/s41467-020-17381-4 (PMC7371868; doi:10.1038/s41467-020-17381-4)
Supplement: Supplementary file 1 — Supplementary Information [file 41467_2020_17381_MOESM1_ESM.pdf]

Supplementary Information

Observation of the geometric phase effect in the $H+HD \rightarrow H_2+D$ reaction below the conical intersection

Yuan et al.

Supplementary Note 1 | Experimental Methods

The H + HD → H_2 + D reaction at 2.28 eV is studied on a velocity map imaging (VMI) - crossed molecular beam (CMB) apparatus[1,2]. Besides the scattering chamber (chamber I), the apparatus is also equipped with two differentially pumped source chambers: one is fixed (chamber II), and the other one is rotatable (chamber III). In this experiment, the pure HI molecules are supersonically expanded by a pulsed Parker valve (Series 9, vertically mounted in chamber II) at the backing pressure of 1 bar. After the expansion, the HI molecules are dissociated by a linearly polarized laser at 213 nm (30 mJ/pulse) at the point about 10 mm upwards the nozzle of the pulsed valve (as shown in Supplementary Figure 1). The photodissociation laser at 213 nm is generated by using the 5th harmonic of an Nd: YAG laser (Continuum, Powerlite DLS 9020). The 5th harmonic is realized by the sum frequency of the fundamental output (1064 nm) and the 4th harmonic (266 nm) of the Nd: YAG with a BBO crystal. At this wavelength, H + I($^2P_{3/2}$) and H + I($^2P_{1/2}$) dissociation channels of HI molecule are both energetically allowed, which give rise to two sharp and separated peaks in the velocity distribution for H atom products, with faster or slower velocities [2]. Due to the totally different angular distributions of the H atom in these two channels, in this experiment, the faster H atom at a speed of 23.00 km/s from H + I($^2P_{3/2}$) channel is selected by setting the photolysis laser to be vertically polarized. The supersonic HD (97% purity, Spectra Gases Inc) molecular beam is generated through a cooled pulsed valve (Even-Lavie valve, horizontally mounted in chamber III) at the backing pressure of 13 bar. The pulsed valve is cooled by liquid nitrogen to further increase the quality of the HD beam and improve the resolution of the experiment. After cooled, the HD molecules expand at a speed of 1.24 km/s (v/Δv ~ 30) and about 97% of the HD molecules are populated in the rovibrational ground level (v = 0, j = 0). The rotational state population of HD beam is measured with the (3 + 1) resonant enhanced multiphoton ionization (REMPI) method around 285.6 nm via the HD ($C^1\Pi$, v =3 ← $X^1\Sigma^+$, v=0), R (0) and R (1)) transitions. The pulsed H atom beam is collimated by a skimmer, and the HD molecular beam is collimated by double skimmers before intersecting in chamber 3 (see Supplementary Figure 1). In order to study the H + HD reaction at 2.28 eV, the crossing angle of H and HD beams is set to be 160°. The experiment is performed at the repetition rate of 20 Hz.

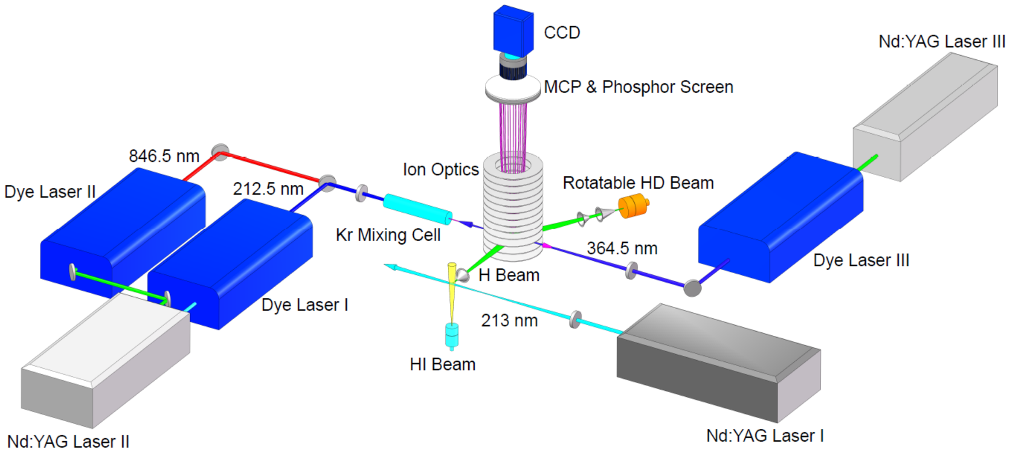

Supplementary Figure 1| Schematic of the experimental setup for high-resolution crossed beams studies in this work.

In the reaction, after the collision of the H and HD molecular beams, the D product channel is selectively detected by using the two-color (1+1′) (vacuum ultraviolet (VUV) + ultraviolet (UV)) REMPI method near the threshold. As shown in Supplementary Figure 1. The VUV excitation laser light at 121.6 nm is obtained by the non-linear four-wave mixing ($2\omega_1$ - ω_2) in krypton[3]. The laser light at ω_1 (212.5 nm) is produced by frequency doubling the fundamental output of a tunable dye laser (Sirah, Cobra-Stretch), pumped by the 355 nm output of Nd:YAG (Continuum, Powerlite DLS 9020). In the four-wave mixing process, the energy of $2\omega_1$ is resonant with the transition between 5p and 4p energy levels of krypton. The fundamental frequency of the other tunable dye laser (Sirah, Cobra-Stretch) is used to produce ω_2 (846.5 nm), which is pumped by the 532 nm output of the same Nd:YAG laser. The UV ionization laser light at ω_3 (364.5 nm) is produced by the frequency doubled output of a third dye laser (Sirah, Cobra-Stretch) which is pumped by another Nd:YAG laser (Continuum, Surelite II). The generation of ω_1 and ω_3 is enabled with BBO crystals in the frequency doubling processes.

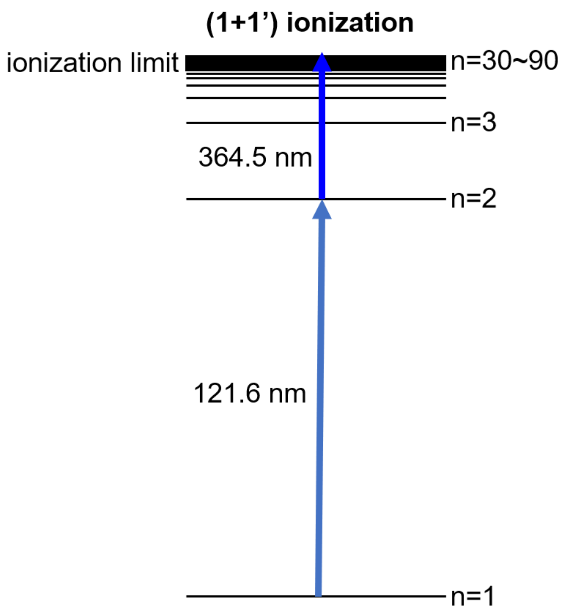

Supplementary Figure 2| The D atom energy levels in the ionization process.

During the (1+1′) REMPI process, the D atom produced in the reaction are excited from n = 1 to n = 2 state by absorbing a VUV photon at 121.6 nm, and sequentially ionized near threshold by absorbing a UV photon at 364.5 nm (see Supplementary Figure 2). The influence of the electron recoil on the velocity of the D^+ is greatly reduced (less than 5 m/s) in this near-threshold ionization scheme. The D^+ ions are accelerated and guided into a field-free time-of-flight (TOF) tube by the ion optics with multiple electrodes [4]. After flew through the TOF tube, the ions are collected by a position-sensitive dual microchannel plate (MCP) with a diameter of 70 mm. The time-sliced measurement is performed by applying a pulsed voltage with a period of 20 ns to the MCP. The time-sliced ion signals from the position sensitive MCP are converted to visible light signals by a phosphor screen (P43) coupled to the MCP. The visible light signals are then captured and recorded as an image by a charge-coupled device (CCD) camera (LaVision pro plus 2M). During the data acquisition, a real-time ion event counting method is applied to further increase the resolution of the ion image. Since the Doppler shift caused by the velocity distribution of the products is beyond the line width of the VUV laser used in this study, in order to detect the products with equal efficiency on the whole velocity scale, the wavelength of the VUV laser is tuned uniformly to cover the Doppler shift of the products by scanning the ω_2 back and forth.

The experimental image in Fig. 1 in the main text shows the full angle differential

cross section (DCS) of the D atom product. In the manuscript, the $\theta = 0°$ and $180°$ are defined as the forward (labeled as “F” in Fig. 1) and backward scattering (labeled as “B” in Fig. 1) directions of the H_2 product respectively, with respect to the direction of the incoming H beam. The resolution of the experiment is high enough that a series of concentric rings were resolved in the experimental image (see Fig. 1 in the main text), denoting the discrete velocities of D atom products. According to the conservation of total momentum, these ring structures correspond to rovibrational states of the H_2 coproduct.

Supplementary Note 2 | Data Analysis

The calibration of the VMI system is carried out with the multiphoton dissociation and ionization of O_2, which was widely used previously[4]. In this study, the measured D^+ signals in the raw image are proportional to the number density of the D products within the detection volume at the time the detection lasers arrive. Since the pulsed and well collimated detection lasers and molecular beams are used in this CMB experiment, the number density of the product is dependent on the velocity of the product. Therefore, in order to eliminate the velocity discrimination in the detection, a density to flux transformation with the experimental parameters considered is performed to derive the corrected experimental image shown in Fig.1.[2]

As stated above in Supplementary Note 1, the resolution of the experiment is high enough to resolve a series of discrete concentric rings in the experimental (as shown in Fig. 1), corresponding to the rovibrational quantum states (v', j') of the H_2 products. By taking a profile of the image starting from the center of mass and over the maximum Newton circle corresponding to the velocity of product H_2 ($v' = 0$, $j' = 0$), the product speed distributions are derived. By applying the momentum and energy conservation laws, the product total translational energy distributions ($P(E_T)$ distribution) are acquired. The product translational energy distribution in the forward scattering direction is presented in Supplementary Figure 3.

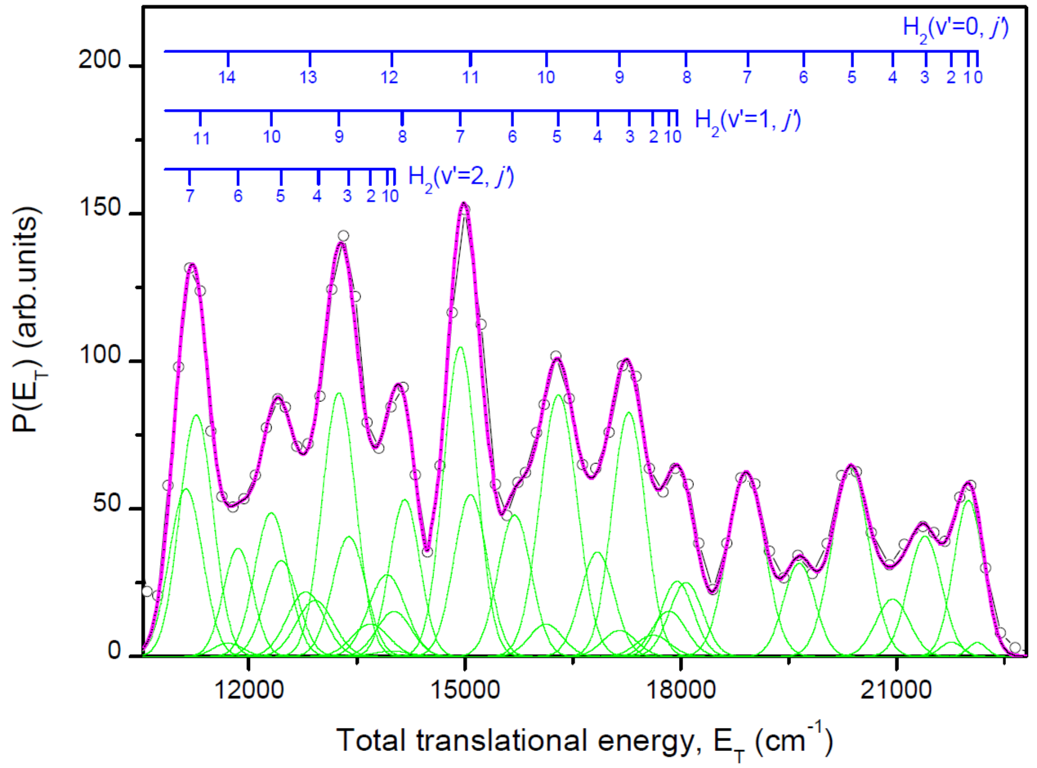

Supplementary Figure 3| Product translational energy distribution of the D atom products at $\theta = 0°$.

By fitting the ro-vibrational states with Gaussians using the accurate energy term values of the rovibrational levels of H_2 in the translational energy distribution at a series of angles, the angular distributions or DCSs of H_2 products can be obtained. The two most pronounced rings shown in Supplementary Figure 3, are labelled as H_2 ($v' = 0$, $j' = 11$ & $v' = 1$, $j' = 7$) and H_2 ($v' = 1$, $j' = 9$ & $v' = 2$, $j' = 3$) by yellow arrows in Fig. 1. The DCSs of these two rings around the forward scattering direction are shown in Fig. 2 in the main text. As shown in Supplementary Figure 3, in the forward scattering direction, the H_2 ($v' = 0$, $j' = 11$ & $v' = 1$, $j' = 7$) and H_2 ($v' = 1$, $j' = 9$ & $v' = 2$, $j' = 3$) states are very intense and well resolved. It enables the high accuracy and reliability when extracting the state resolved DCS by fitting the $P(E_T)$ distribution. For the DCSs shown in Fig. 2, the measurement error (due to counting statistics) in the current experiment is about 3% (Standard Deviation divided by the average valve, SD/AVG)) in the forward scattering direction due to the good stability of the experimental apparatus.

Supplementary Note 3 | Quantum reactive scattering theory including the GP effect

One way to include the GP is to calculate the multivalued wave functions of electrons and nuclei, which satisfy the following equations

$$\Phi_e(\eta + 2\pi) = -\Phi_e(\eta) \qquad (1)$$

$$\Psi_n(\eta + 2\pi) = -\Psi_n(\eta) \qquad (2)$$

where phase angle η can be any value that $\eta \rightarrow \eta + 2\pi$ describes a closed path around the conical intersection (CI). This method requires that Ψ_n to be expanded with a multivalued basis set, i.e., the functions of which must change sign on encircling the CI.

Although such a basis set could be implemented with the hyperspherical coordinate, it is applicable only for the reaction of H + H_2 whose the CI locates at the origin of the coordinate. In the mass-scaled hyperspherical coordinate, the CI for the H + HD system does not locate at the origin of the coordinate system, which does not allow a convenient implementation of the double-valued basis functions for describing nuclear motion.

The other way, which is of general applications, for including the GP is to multiply the real double-valued electronic wave functions by a complex phase factor such that the new electronic wave functions are complex single-valued functions around the CI. Correspondingly, the nuclear wave functions are now single-valued functions around the CI. This is the vector potential approach introduced by Mead and Truhlar[5]. Following previous work, a phase factor of the form

$$\Phi_e \rightarrow \exp\,(i\frac{l}{2}\eta)\Phi_e \qquad (3)$$

where l must be odd to ensure Φ_e single-valued. Accordingly, the nuclear Laplacian operator is modified according to

$$-\nabla^2 \rightarrow (-i\nabla - \boldsymbol{A}) \cdot (-i\nabla - \boldsymbol{A}) \qquad (4)$$

where the vector potential $\boldsymbol{A}$ is given by

$$\boldsymbol{A} = -\frac{l}{2}\nabla\eta \qquad (5)$$

Using equation (4) to incorporate $\boldsymbol{A}$ into the Hamiltonian, the method of vector potential yields a single-valued nuclear wave function Ψ_n.

In this way, it is convenient to incorporate the GP into a standard adiabatic non-GP state-to-state wave packet calculation, since the same coordinate system and grid basis functions may be used.

Different from our previous work[6], in this work the hyperspherical coordinates is applied in the state-to-state calculation using the time-dependent wave packet method. The details of the method for simple adiabatic reaction dynamics have been presented in Ref. 7. To include the GP in this method, one can accomplish by applying equation (4) and adding the extra terms that result to the Hamiltonian. Since the Hamiltonian operators have been derived in the work by Kendrick[8], we do not repeat here.

As mentioned above, the phase angle η can be any form that $\eta \rightarrow \eta + 2\pi$ describes a complete path round the CI. Using the hyperspherical coordinate, the general phase angle η for the isotopologues of the H + H_2 reaction can be written as

$$\eta = \frac{\cos\frac{\theta}{2}\sin\phi}{sin\frac{\theta_0}{2}\cos\frac{\theta}{2}cos\phi + cos\frac{\theta_0}{2}sin\frac{\theta}{2}} \qquad (6)$$

where the way to calculate the value of θ_0 has been given in the work by Xu et al.[9] and d is dimensionless scaling factors.

The vector potential components $(\boldsymbol{A}_R, \boldsymbol{A}_r, \boldsymbol{A}_\Theta)$ can be obtained straightforward by calculating the first derivative of η. For calculating the state-to-state *S*-matrix elements, the initial and final wave packets are changed according to

$$\Psi(\rho, \phi, \theta; 0) \rightarrow \exp\left(-i\frac{l}{2}\eta\right)\Psi(\rho, \phi, \theta; 0) \qquad (7)$$

$$\Psi(\rho, \phi, \theta; f) \rightarrow \exp\left(-i\frac{l}{2}\eta\right)\Psi(\rho, \phi, \theta; f) \qquad (8)$$

In the calculation of the DCS up to collision energy 4.0 eV, partial waves with total angular momentum up to $J = 60$ are calculated. The numerical parameters applied in the calculations are listed in Supplementary Table 1, which has been well tested for obtaining converged results.

The second order split operator is applied in our work to realize the time propagation. In the NGP calculations, it is noted that the Born-Huang term, or the diagonal non-Born-Oppenheimer correction (DBOC) has been included in the calculations.[10,11]

Supplementary Table 1| Numerical parameters used in the quantum reactive scattering wave packet calculations. (atomic unit was used unless otherwise stated)

Grid range and size	$\rho \in [0.01, 17.0]$, $N_\rho = 199$
	$\phi \in [0, \pi]$, $N_\phi = 256$
	$j_{min} = 0$, $j_{max} = 70$, $N_j = 71$
Initial wave packet	$E_0 = 1.8$ eV, $R_0 = 8.0$, $\delta = 0.2$
Matching plane	$R^\infty = 7.0$
Absorbing potential	$n = n' = 2$, $C_\rho^1 = 0.04$, $C_\rho^2 = 0.2$, $\rho_1 = 11.5$, $\rho_2 = 16.5$
K-block	min (J+1, K_{max}=30)
Total propagation time	3000 iterations with $\Delta_t = 2.0$

References

1. Yu, S. R., Yuan, D. F., Chen, W. T., Yang, X. M. & Wang, X. A., VUV Photodissociation dynamics of nitrous oxide: The O($^1S_{J=0}$) and O($^3P_{J=2,1,0}$) product channels, *J. Phys. Chem. A* **119**, 8090-8096 (2015).
2. Yuan, D. F.et al. Direct observation of forward -scattering oscillations in the H+HD→H_2+D reaction, *Nat. Chem.* **10**, 653-658 (2018).
3. Hilbig, R. & Wallenstein, R., Narrowband tunable VUV radiation generated by nonresonant sum- and difference-frequency mixing in xenon and krypton, *Appl. Opt.* **21**, 913-917 (1981).
4. Lin, J. J., Zhou, J. G., Shiu, W. C. & Liu, K. P., Application of time-sliced ion velocity imaging to crossed molecular beam experiments, *Rev. Sci. Instrum.* **74**, 2495-2500 (2003).
5. Mead, C. A. & Truhlar, D. G., On the determination of Born–Oppenheimer nuclear motion wave functions including complications due to conical intersections and identical nuclei, *J. Chem. Phys.* **70**, 2284 (1979).
6. Yuan, D. F. et al. Direct observation of the geometric phase effect in the H+HD→H_2+D reaction, *Science*, **362**, 1289-1293 (2018)
7. Zhao, H. L., Hu, X. X., Xie, D. Q. & Sun, Z., Quantum wavepacket method for state-to-state reactive cross sections in hyperspherical coordinates, *J. Chem. Phys.* **149,** 174103 (2018)
8. Kendrick, B. K., Geometric phase effects in the H+D_2→HD+D reaction, *J. Chem. Phys.* **112,**

5679-5704 (2000)

9. Xu, Z. R., Baer, M. & Varandas, A. J. C., On phase factors and geometric phases in isotopes of H_3: A line integral study, *J. Chem. Phys.* **112**, 2746-2751 (2000).
10. Mahapatra, S., Köppel, H. & Cederbaum, L. S., Reactive scattering dynamics on conically intersecting potential energy surfaces: The H + H_2 exchange reaction, *J. Phys. Chem. A* **105**, 2321-2329 (2001)
11. Bouakline, F., Althorpe, S. C. & Ruiz, D. P., Strong geometric-phase effects in the hydrogen-exchange reaction at high collision energies, *J. Chem. Phys.* **128**, 124322 (2008).
