# Supplementary material for: Observation of the geometric phase effect in the H+HD→H2+D reaction below the conical intersection
Source: Nat Commun. 2020 Jul 20;11:3640. doi: 10.1038/s41467-020-17381-4 (PMC7371868; doi:10.1038/s41467-020-17381-4)
Supplement: Supplementary file 2 — Description of Additional Supplementary Files [file 41467_2020_17381_MOESM2_ESM.pdf]

Description of Supplementary Files

File Name: Supplementary Movie 1

Description: The representative trajectories for the forward scattering of the H + HD → H2 (v′ = 2, j′ = 3) + D reaction.
