# Supplementary figures and images for: Observation of the geometric phase effect in the H+HD→H2+D reaction below the conical intersection
Source: Nat Commun. 2020 Jul 20;11:3640. doi: 10.1038/s41467-020-17381-4 (PMC7371868; doi:10.1038/s41467-020-17381-4)

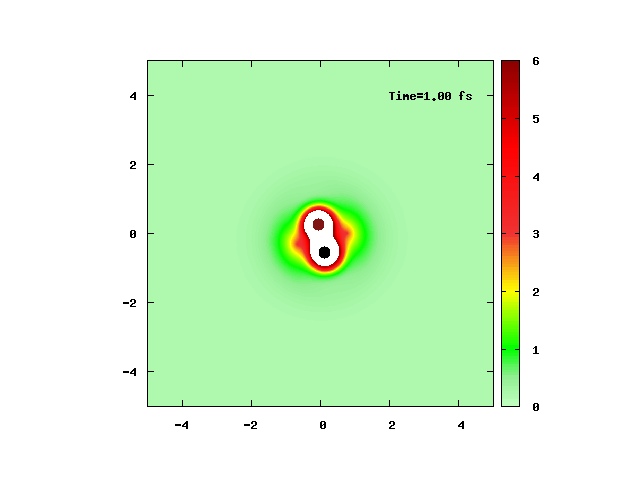

Supplement: Supplementary file 3 — Supplementary Movie 1 [file 41467_2020_17381_MOESM3_ESM.gif]
